# Public perception of drinking water from private water supplies: focus group analyses

**DOI:** 10.1186/1471-2458-5-129

**Published:** 2005-12-09

**Authors:** Andria Q Jones, Catherine E Dewey, Kathryn Doré, Shannon E Majowicz, Scott A McEwen, David Waltner-Toews, Spencer J Henson, Eric Mathews

**Affiliations:** 1 Division of Community Health, Faculty of Medicine, The Health Sciences Centre, Memorial University of Newfoundland, St. John's, Newfoundland, A1B 3V6, Canada; 2 Department of Population Medicine, University of Guelph, 50 Stone Road East, Guelph, Ontario, N1G 2W1, Canada; 3 Foodborne, Waterborne and Zoonotic Infections Division, Public Health Agency of Canada, 160 Research Lane, Suite 206 Guelph, Ontario, N1G 5B2, Canada; 4 Department of Agricultural Economics and Business, University of Guelph, 50 Stone Road East, Guelph, Ontario, N1G 2W1, Canada; 5 City of Hamilton Health Protection Branch, Public Health and Community Services, 1 Hughson Street North, Hamilton, Ontario, L8R 3L5, Canada

## Abstract

**Background:**

Over four million Canadians receive their drinking water from private water supplies, and numerous studies report that these supplies often exceed the minimal acceptable standards for contamination. Canadians in rural areas test their water intermittently, if at all, and treatment of water from private supplies is not common. Understanding the perceptions of drinking water among residents served by private systems will enable public health professionals to better target education and outreach activities, and to address the needs and concerns of residents in their jurisdictions. The purpose of this study was to explore the drinking water perceptions and self-described behaviours and needs of participants served by private water systems in the City of Hamilton, Ontario (Canada).

**Methods:**

In September 2003, three focus group discussions were conducted; two with men and women aged 36–65 years, and one with men and women 20–35 years of age.

**Results:**

Overall, participants had positive perceptions of their private water supplies, particularly in the older age group. Concerns included bacterial and chemical contamination from agricultural sources. Testing of water from private supplies was minimal and was done less frequently than recommended by the provincial government. Barriers to water testing included the inconvenience of the testing process, acceptable test results in the past, resident complacency and lack of knowledge. The younger participants greatly emphasized their need for more information on private water supplies. Participants from all groups wanted more information on water testing, and various media for information dissemination were discussed.

**Conclusion:**

While most participants were confident in the safety of their private water supply, the factual basis for these opinions is uncertain. Improved dissemination of information pertaining to private water supplies in this population is needed. Observed differences in the concerns expressed by users of different water systems and age groups may suggest the need for targeted public education strategies. These focus groups provided significant insight into the public perception of private water supplies and the need for public health outreach activities; however, to obtain a more representative understanding of the perceptions in this population, it is important that a larger scale investigation be performed.

## Background

Over four million Canadians receive their drinking water from private water supplies, predominantly from groundwater wells [1]. Numerous studies report that Canadian private water supplies often exceed the minimal acceptable standards for microbial and chemical contamination [1-5], and it is estimated that 45 percent of all waterborne disease outbreaks in Canada involve non-municipal systems, largely in rural or remote areas [1]. Further, several studies report that Canadians in rural areas test their water only intermittently, if at all, and that treatment of water from private supplies is not common [1-3]. Understanding the perceptions of drinking water among residents served by private water systems will enable public health professionals to better target public education and outreach activities, as well as address the needs and concerns of residents in their jurisdictions.

Several surveys of drinking water consumption behaviour in North America have been performed [2,6-10], some of which explore the reasons for alternative water use, such as bottled water and water treated with in-home devices [6-9]. However, these studies mainly focus on municipally treated water, and are quantitative/semi-quantitative in nature, providing only a general understanding of residents' perceptions. Focus groups, as with other qualitative methods, are useful in generating rich, detailed, data that cannot be acquired via the use of quantitative surveys, and allow for in-depth exploration of participants' attitudes and responses [11,12].

The purpose of this study was to explore, in-depth, the drinking water perceptions and self-described behaviours and needs of participants served by private water systems in the City of Hamilton, Ontario (Canada). This included participant perceptions of drinking water, their perspectives and behaviours with respect to water testing, the reasons behind any alternative water use and their self-identified needs and desire for information pertaining to private drinking water supplies.

## Methods

The City of Hamilton is a large urban centre surrounded by suburban and rural areas. In September 2003, we performed three focus groups with English-speaking, adult residents (20 years and older) of the City who received their household water from private (i.e. non-municipal) water supplies, including private wells and cisterns. To identify residences with private water supplies for recruitment, we linked residential addresses to digitized maps of the distribution areas served by the City's water treatment utilities within a Geographic Information System (ArcView GIS 3.1, Environmental Systems Research Institute Inc). Residences not falling within municipal water polygons were classified as having a private water source and were included in the sampling frame. These addresses were then cross-referenced with a commercial database of residential telephone numbers for the City of Hamilton. We developed recruitment criteria that were used by a professional marketing firm for telephone screening and enrollment of participants. To gather information from residents of various ages and to avoid problems with mixing distinct age groups, we stratified the focus groups by age. Two focus groups were conducted with men and women between 36 and 65 years of age, and one with men and women between 20 and 35 years of age. Exclusion criteria included being employed in the water industry and having participated in a focus group within the last calendar year. Eight or nine participants were recruited for each focus group to ensure attendance of at least six participants per group [11,12]. An exception to this was the focus group conducted with participants between 20 and 35 years of age, for which only six participants were recruited. Participants were provided a small honorarium for their participation. The Human Subjects Committee at the University of Guelph approved the study and all participants provided written, informed consent.

A trained facilitator moderated the focus group discussions, which were audio-taped and professionally transcribed to maximize data capture and facilitate analyses. An assistant also recorded notes on the discussion and group interactions. A pre-tested, structured questioning route was developed according to Krueger and Casey (2000), using a combination of structured questions and pre-planned probes to improve detail and understanding. The focus group discussions were carefully moderated to gather data regarding participant perceptions of water quality, alternative water use, water testing, and their self-identified need for information pertaining to private water supplies. Systematic procedures were used to help to ensure reliability and validity in data collection, including verifying data with participants during and at the end of each focus group, a debriefing session between the moderator and assistant-moderator immediately after each group, and the use of field notes and audio-transcripts.

Transcripts were checked for accuracy against the original audio recordings and field notes. Major coding categories were derived from the questioning route and sub-themes were derived from content analysis using methods described in the qualitative methods literature [11-13]. Direct quotations from participants were used for support and illustrative purposes; proper names and profane words were removed from the quotations reported herein, and portions of quotations that needed clarifying context were supplemented with additional text that was placed within square brackets.

## Results

### Participants

Five people attended the first of the two focus groups conducted with people between 36 and 65 years of age, and six attended the second. Four people attended the focus group conducted with participants between 20 and 35 years of age. Each group had a roughly equal proportion of men and women and reflected a variety of income levels and educational backgrounds. All participants were Caucasian.

### Perceptions

The facilitator asked very general questions to stimulate discussion regarding the participants' perceptions of their drinking water. Without direct influence from the investigators, participants introduced into discussion several broad themes, each of which is discussed in turn below:

#### Sensory quality of water

Participants' perceptions of the sensory quality of drinking water from their private supplies were overwhelmingly positive, particularly within the 36 to 65 year-old age group. The majority of participants reported their water to be "excellent" in taste. Common words used by participants to describe their water included "great tasting", "fresh", "very cold", "no chlorine" and "no smell". Participants reported only two distinct troubles with the sensory quality of their water. Many commented on the hardness of the water and disliked the effect it had on appliances and plumbing. A few participants also said they disliked the sulphur smell and taste of their water ("when we moved to our house it was sulphur. It was quite sulphur. I couldn't get my taste buds around it..."). However, participants generally referred to the hardness and sulphur content of their water as "inconveniences", rather than concerns.

#### Water safety

Most participants, particularly within the 36 to 65 year-old age group, were very confident in the safety of their water; all of the participants in one group even reported having "no concerns at all". Participants gave two main reasons for their belief in the safety of their water. One related to the independence of private water systems; not having to rely on others for the provision of their drinking water. Two participants illustrated this theme well when they explained why they perceived their water as safe:

" [In the city], there's a lot of things you depend on, whereas... out in the country at least you have that independence. I know where my water is coming from. I know what's in it. I don't have to worry about someone monkeying with it."

"... partly because if you've got a well on your property, it's your responsibility to take care of it, so you *know *who's looking after it. That makes it pretty darn safe."

The following participant statement was also met with high agreement:

"...there's a sense of that independence too and not having to depend on someone else to chlorinate the water, to make sure the system works."

The well-publicized *E. coli *0157:H7 outbreak, associated with the inadequate management of municipal well water in Walkerton, Ontario in 2000 [14], was often raised in support of this point.

The natural filtration associated with ground water supplies was the other main reason for the participants' confidence in the safety of well water. One participant expressed this as follows:

"...by the time [contamination] seeps into your well, everything has been filtered out. The water, the earth is a natural filter, and you just get cleaner water...".

Despite confidence in the safety of their water supplies, several participants discussed the potential for contamination of their water with pesticides, fertilizers and fecal run-off from nearby farms. One participant said:

"... one of the things that we always have our heads up with, is because we're country, and there's a huge pig farmer and a dairy, a cow farmer beside us... They have open pits and every spring they spread over the fields before they plant them, they spread and that actually at the end of the day becomes groundwater."

She went on to add however: " [But] like really, it's well filtered. We have a 150 foot well too."

Two participants mentioned concerns that their children would likely be the first family members affected by contamination of their water source. For instance, one commented:

"But I do worry more about [contamination], probably as any parent would, like, because I have a small child, so I think when your children are small, well, I don't worry as much for myself; I probably should, but I think you get more concerned...".

While participants in the 36–65 year-old age group agreed that contamination from agriculture was a possible health risk, several also said that it was inherent with country living and "just something to think about". Overall, there was a tremendous sense of pride and contentment in the quality and safety of water from private water sources in this age group.

Confidence in the safety of well water was not as pronounced among participants in the 20–35 year-old group. One participant explained having concerns about the safety of her well water because of a crack in her well:

"I know there's a leak in there. Whatever can get out can come in. You know, and ...I live on a cow farm, so I do not touch my [well] water."

Another participant, although greatly appreciative of his well water, said:

"I guess you're always kind of worried about something seeping into it, right?"

When asked to explain, he replied:

"Just like, stuff like Walkerton, like you hear *E. coli *and stuff like that and you never really know what's being dumped in the ground... like if no one's watching... dumping chemicals or something because that's going to go directly into your water."

These two participants also had views regarding self-sufficiency that contrasted with those of the other participants. For instance, one participant spoke of municipal water:

"At least the city has someone designated to supposedly be watching it, right? So there should be some kind of, um, supervising element to it, whereas the well water, you just, you're relying that it's good...".

The other participant with concerns then agreed: "Nobody's checking it out", to which the first replied: "You're never really 100% sure."

Despite this, all participants, with exception of the one group member with the crack in her well casing, considered their water to be "just as safe" or "safer" than municipal water. They reported a number of reasons for this perception, including: the "natural filtration of wells", the high probability of any contaminants being diluted to safe levels, their water not having to pass through "dirty" or failing municipal distribution systems, and the lower likelihood of their water being a target for terrorist attack.

#### Effect of rural development on the water aquifer

The most extensive concern was that nearby development and construction would have a negative effect on the water aquifers supplying the participants' wells. Two illustrative participant statements included:

"The only concern we had was the new development they were putting up in behind us, whether it's going to screw up things and alter the water quality. Every time they do a major development over there you sort of check..."

"I'm fortunate, I really don't have any development around me, I just have one neighbour and uh, no sign of anything going in, so I know that I'm safe for a while anyways. There's no developments going in, but you never know."

#### Concerns pertaining to specific types of private water systems

Several participants expressed concerns about the costs of replacing water pipes, especially given that these costs are incurred by themselves. One participant said that he worried about the expense and inconvenience of alternate water sources should the water from his well no longer be available.

Another participant whose house was supplied by a water cistern was annoyed with insufficient quantities of water. On having a cistern she said:

"... it's just a pain in the neck if you run out of water. I mean, I could be in the middle of a shower and all of a sudden, I have no water."

#### Need for perspective

Several participants commented that there was a need to maintain perspective when considering concerns with drinking water. One participant illustrated this theme well:

"If you were living on a well and you're saying, my God, I wonder what that farmer next door put into my water. You know. Is it going to be safe?... You'd drive yourself crazy. So you actually have to build up some sort of confidence in whatever system you have just to avoid driving yourself insane."

Hence, while some participants recognized the potential risks associated with private water supplies, most reported that they choose not to "dwell" on these concerns.

### Bottled water

With the exception of the participant who relied solely on bottled water because of the crack in her well casing, participants had only negative comments regarding it. Two themes were evident: poor taste and skepticism as to the water source. Regarding taste, one participant said, "it tastes like plastic to me" and several participants agreed with the following participant statement:

"I can't drink that bottled stuff, it's just blah... It quenches your thirst and that's about it, but other than that it's got no taste to it and if I didn't have to drink it I wouldn't."

Many participants were also suspicious that commercial bottlers made fraudulent claims regarding their water sources. The participants' distrust of bottled water was well captured in one participant's comment:

"... these people that buy bottled water all the time, I'm asking, do you know where that came from? [They say] 'It's all right. It's in the bottle.' [I say] what the [heck] has that got to do with it – it's got a cap on it? Don't you remember the scandal of [a prominent water company], when they found it wasn't coming from where it was supposed to be, ten years ago. They were getting it... not from the artesian well that they said they were getting it from, and they proved that [the company] had been scamming [people] for five years. You don't know what's in [bottled] water... You don't know where it's coming from..."

Despite their negative opinions, participants in the 36–65 year-old age group reported occasional consumption of bottled water, with convenience being the most extensive reason for its use. The participants reported using bottled water merely because the bottle was portable, convenient and generally well-accepted in the workplace and schools. One participant said:

"It's really just truly the convenience of having it in the fridge with the lid on it that I can take in the car or to work".

Further, many participants reported re-filling bottles with the water from their private supply. Many also said that they only purchase bottled water when they were out and chose it as a substitute for other beverages. With the exception of one participant who said she preferred bottled distilled water for making coffee, all participants in the 36–65 year-old age group said convenience was the only reason for purchasing bottled water.

In the 20–35 year-old age group however, participants reported two reasons for using bottled water. As with the older participants, there was a preference over other types of beverages while outside of the home. However, concerns were also expressed about the quality of their well water. The participant with a crack in her well reported:

"When we first moved in it was brown, murky... [we] couldn't see through it and we were told that if we boiled it, it would be fine. But when you have a family, you know, it was just a lot easier to... get a cooler and just go with the spring water."

Another participant reported that his family first purchased a bottled water cooler when:

"they were doing quarry and mining and stuff around... And from what I understand, [they] cracked the bedrock and sulphur had leaked into the water, and then I think that's when it started smelling bad".

This household had later installed a treatment device to resolve the sulphur problem, and then discontinued their in-home bottled water use.

### In-home water treatment devices

Most participants used devices to treat the water from their private water supplies; the most common devices were water softeners. A few other systems were reported, including a water distiller and ultra-violet light and reverse osmosis devices. Of the two participants who did not use treatment devices, one was the participant who consumed only bottled water from dispensing coolers in her home. There were two major reasons for the use of treatment devices: to reduce the hardness and sulphur content of well water and to increase the safety of water from cisterns.

The majority of participants reported using water softeners in order to decrease the negative effects of hard water on plumbing and appliances. Many participants shared the set-up described by one participant:

"We use a water softener just because of the hardness, but then we have a three pipe system which is treated hot and cold, and an untreated cold line for drinking."

Some respondents reported using treatment devices because their water had a sulphur smell and taste, which they found disagreeable.

Participants who used water cisterns however, reported using ultraviolet light or reverse-osmosis devices in order to clean and decrease the contamination of their water. One cistern-owner explained their use of a treatment system:

"We just thought [it was] probably safer... our eaves troughs, I mean, they're regularly cleaned, but we just thought it's probably safer for cooking and [consumption]".

Another explained:

"You know, just to feel more confident."

Several well-owners agreed that treatment systems would be necessary for drinking water from cisterns ("Safety wise, yeah, you'd have to"). However, well owners reported that, to the contrary, they used treatment devices to decrease the hardness or sulphur content of the water, and not out of concerns for safety.

### Private water testing behaviours

Participants were asked how frequently they tested the water from their private water source. Some participants tested it once per year and some tested every two to three months. However, many participants reported that they had never tested their water, or only tested it once every few years. A few participants were unsure as to whether their water was tested, but speculated that another member of the household might take this responsibility.

When asked what tests were undertaken on their water, only few participants gave a definitive answer ("*E. coli*"); several were unsure because another member of the household did the testing ("All I know is that [my parents] bottle up a sample and they send it to whatever facility that tests it and we get a report back."). Even among those participants who did test their water regularly, many reported not knowing what the water was specifically tested for. One participant said:

"I send it for testing, and they [at the lab] figure out what they're testing it for."

Overall, the extent of testing appeared to be limited to coliforms, and a general feeling of uncertainty existed across participants with respect to water test parameters.

Respondents who tested their water reported doing so for various reasons. Several said they regularly tested it because of failed tests in the past:

"... when we failed the test, that's when we upped the ante a bit in terms of getting it tested."

Many participants also reported testing their water because they had learned of local water problems, either from neighbours or, in some cases, because of an informative flyer being distributed to their homes. Several participants explained:

"...there was a lot of talk about some of the houses right down in the village having problems, so it was close enough, hearing about local problems, we figured we'll test ours and see what it is."

"Until that point we'd never tested it, since I was a kid, but once we got that flyer, we test every year now."

Respondents gave numerous explanations for not testing their water regularly. Many said the inconvenience of testing prevented them from testing more often. There were many complaints of having to make several trips to the city in order to pick-up and drop-off sample bottles. The following participant statement was met with high agreement:

"It's as I say, two or three trips to some place which you might not necessarily go by. You've got to make a special trip. And when we're talking country people having to get into the city, some of us avoid coming into the city."

Participants also reported not testing their water regularly because their test results in the past had always been negative. The following participant statements were indicative:

"You get a couple of good readings over and over and over again that you've got fantastic water, and after a while I say 'oh, well, it's fantastic water'. Why am I going to waste my time with all this?"

"... we only test our water once a year. Maybe we should do it more, but because it always comes back zero-zero, we just don't do it."

Many participants also reported "complacency" as a reason for not testing their water more often. For instance, several participants who reported never testing their water commented:

"Oh, I'm not all that diligent in checking my water. As long as it tastes good, that's fine."

" [I'm] lazy. I'm not worrying about it enough to do it. When something comes up and people start getting concerned, everyone's testing and complaining, well, [then] we'll get it tested."

Some participants said that infrequent testing was also due to ignorance and not knowing that water testing was available or necessary. One participant explained:

"But see, and I mean, I've lived out there for... years and I didn't realize that there were places that you could take your water to get tested, or whatnot."

Another participant suspected that ignorance was especially the case for "ex-urbanites" who had recently moved to the country. He explained:

"...there are a lot of people who move from the big city out into the country, and don't realize they should be testing their water... I know a couple of people who've moved in [to rural areas] recently and they've never heard of testing water, because they come from a [municipal] system: you turn the tap on, it's there."

A few participants also reported not testing their water because of fear of the "government's" response to a positive result:

"Once you put your water in to be tested, then they expect you to fix it if it's broken".

Another participant agreed that this would deter people from testing their water:

" [Especially] if it was a rental property... it's almost like you're inviting more expense to yourself if they find something."

### Methods to encourage private water testing

The participants said that public education would help to increase water testing among people with private water supplies. In some instances, it was seen as serving to provide new information, in others it would act as valuable reminders that would "at least get us thinking about it more". An idea that garnered a lot of support in all of the groups was an educational campaign that made regular water testing habitual. Several participants likened this to the popularized North American reminder to change batteries in smoke alarms when clocks are changed for Daylight Savings time:

"It's like, you always hear about it and you think, okay, change your clock, you think about your [smoke alarm] battery. It becomes, it's like advertising or something... it becomes part of your consciousness over time."

Many participants thought that one-time reminders would not generate sustainable change; instead, water testing needed to become an engrained action that was part of rural-life culture.

Another extensive theme to encourage water testing revolved around making the testing process more convenient. Many participants said that they would appreciate having a pick-up service, where someone, preferably students doing community work, would come to their house to collect water samples for testing. One participant illustrated his reasoning:

"I would like a student to come by and test every household. [Someone] with a bottle for water, saying... 'I'm with the City of Hamilton. We're running this program. We're going to test your water and we'll send you back your results.' Because you're going to get older people. You're not going to teach an old dog new tricks. You can talk until you're blue in the face. Until someone does it for them, they won't do it."

Another participant said that she thought it would be very effective because:

"... we are lazy, everybody is. We've got our lives, we're all busy, you know. You go home, you just want to take off your shoes and get the converter, lay on the couch, you know. But [with such a service] you're more apt to do it, I think."

Other participants agreed that the process of water testing should be made more convenient, possibly by increasing the number of pick-up and drop-off locations, particularly in the more rural areas:

"I mean, in a little place like [a village within the City], if they had a drop off place where you picked up your mail, and say, they picked a day and they said, okay, the fifth of the month is water testing day. You drop off your bottle for the fifth of the month, someone from the lab comes by and picks up the carton of water from local people who want it taken, you might do it more often."

Some participants also said that they would like a home test kit, which would make the process most convenient. Finally, many participants thought that having access to test results from other wells near to their home would dramatically encourage testing of water from private supplies. There was generous support for an accessible system that would share private water test results, ideally through a mapping system:

"Like, the idea of a map saying the water quality has been tested here and here and here. Here are the areas with some problems, these are areas that are doing well."

The participants emphasized the need for confidentiality with such a system, but also said that it would be an effective way to encourage regular water testing. As noted previously, many participants had tested their water in response to learning of water problems in neighbouring areas.

### Public education

Participants were asked whether they wanted to receive information regarding water from private water supplies. Although most participants concurred with this, two members from the 36 to 65 year-old groups said that they did not need nor want any further information, because they were confident in the safety of their wells. Another participant said, "I wish I knew more", but had concerns about being overloaded with information.

As noted above, many participants wanted a system that would share water test results from surrounding areas. The request for this information was repeatedly illustrated in all of the groups. Many participants also indicated a desire for more information pertaining to water testing. Specifically, they wanted to know what test parameters were available in the free test supplied by the City, what other tests were available and should be performed, the costs of these tests and the laboratory that performed them. Several participants said that they would like this information, but would like it specifically customized; for instance what their water should be tested for given their particular geographic location:

"I would like to know... what else I could have my water tested for, and maybe should have my water tested for, depending on the area I live in."

Participants also said that they would like water test results explained in clear language, specifically indicating what effects different components of their water might have:

"Like, learning the different components of water and what's good, what's bad, I mean, so you have a lot of sulphur in your water. Is that going to harm you?"

Several participants also wanted information pertaining to water treatment options, based on any problematic water test results they might receive. Finally, a few participants said that they would appreciate general waterborne disease information, including what pathogens may be in their water and what illnesses could result.

In the 36–65 year-old age group, there was a recurring theme pertaining to education. Several participants spoke of the need for knowledge and respect for private well water systems, including their planning, construction and maintenance. They said that many negative health events associated with well water were because this mind-set was lacking. Examples included contamination associated with running stale wells or city layouts where shallow wells were located downhill from agricultural farms. One participant spoke of several areas in Southern Ontario where:

"all these farms sit above and the town sits down below and you start having shallow wells. [Contamination is] what's going to happen with ground run-off. You can't get away with it."

The overall impression these participants gave was the need for well owner education with respect to proper well planning, construction and maintenance.

There were clear differences between the two age groups in the intensity of participants' self-identified need for information. Participants in the 36–65 year-old age group were largely content with their current level of general knowledge, but wanted more information in specific areas, like testing and test results. In the 20–35 year-old age group however, participants stated that they were deeply lacking in general knowledge, and greatly emphasized their need for information. One participant explained:

"I mean, I've lived out there for... years and I didn't realize that there were places that you could take your water to get tested, or whatnot. So the awareness is definitely not there. ...If I don't want to get off my lazy [behind] and go and do [the testing], that's *my *choice. [But] right now, I didn't know I *had *a choice."

Another participant said he wanted: "just generally more [information], you know", but had difficulty explaining what he specifically wanted: "See, I don't know what I need to know, so I don't know what I want to know..."

Among all groups, a wide range of media was suggested for disseminating information. Common responses included local newspapers, flyers/brochures mailed to the home, a city webpage, and radio messages played on local stations during commuting hours. A few participants also suggested short, informative television "commercials". Participants also offered advice with respect to designing effective educational programs. Many said that the information should not be presented in such a way as to cause people alarm or panic: "You see, you've got to be *so *careful that you don't start scare-mongering." While the majority of participants supported this, one stated that some element of fear was required to motivate people to read or use the information:

"...that element of fear had to happen before we even worried about our water and I think that's what's needed to kick people in the rear end to test their wells".

Participants also said they wanted something direct, and to the point:

" [If I take] a quick glance and it's to the point, 'people's well water, bang, see this website', that would grab my eye. It would only take me five seconds and I'd say well, that might be interesting. I better check that later."

Many participants thought the flyers should include only the basic facts and should direct people to another source (for example, a website or other publication) for more information:

"Don't put a lot of detail on it, just put where you could find the information that you should be looking for".

With respect to information disseminated via the internet, some participants said that they wanted a direct link to information so they did not have to navigate through a larger web page.

## Discussion

Most participants in this study expressed a strong appreciation and a high degree of confidence in the safety of their private water supplies. Many had trust in the safety of their water despite not having had it tested or using a treatment device. Several studies in Ontario and other parts of Canada however, have shown chemical and microbial contamination of private water supplies in excess of government standards for safe drinking water [1,3,4,15,16], and similar results have been observed in other developed countries [17-19]. Some participants were concerned that nearby agricultural operations and building development could contaminate their wells or otherwise have a negative effect on their aquifers. However, these participants seemed to be concerned only if these activities were occurring immediately nearby their homes. With the exception of one participant, most did not suggest that contamination of aquifers might occur some distance from the wells themselves. Further, given that some waterborne illnesses are self-limiting and/or require chronic exposure, waterborne hazards may be present without the owner's recognition, thereby posing a risk to residents of the household. Also, because residents may eventually develop immunity to a pathogen(s) present in their water, a hazard may go unrecognized and pose risk to visitors to the household. Thus, the inherent confidence of the focus group participants in the safety of their water supply may or may not be warranted; many of the participants did not have adequate information or test results on which to soundly base their opinions. If these perceptions are representative of the general population, it could indicate the need for increased dissemination of information, including possible water contaminants and their effects, the importance of regular testing of private water supplies, the fact that development or agriculture does not necessarily need to be very close in proximity to affect well water quality, and that residents relying on private water wells in Ontario have a legal responsibility for the condition of their wells.

Participant concerns included bacterial and chemical contamination from agricultural sources, and in the 20 to 35 year-old group, illegal dumping of pollutants and the lack of an official monitoring system for drinking water from private water supplies. Identification of drinking water concerns in the larger population might help policy makers ensure that their activities and attention include the areas of their constituents' concerns.

Bottled water use among participants in the 36 to 65 year-old age groups was not common, and participants reported using it solely for convenience or as a substitute for other beverages while not at home. Two participants in the 20 to 35 year-old age group reported choosing bottled water because of concerns regarding the safety or sensory quality of their well water. A survey of municipal water consumers in the U.S. reports that people between the ages of 18 and 34 years are more likely to believe in the safety and health benefits of bottled water compared to those over the age of 35 [9]. Generational differences in the degree of concern and interest regarding water may explain the differences between the two age groups and such differences could have implications for the targeting of public education strategies.

Perceptions also differed between the two types of private water systems discussed, namely wells and cisterns. Participants who received water from cisterns reported using treatment devices because of contamination concerns. The majority of well owners however, used only water softeners, while leaving the cold water line in the kitchen untreated. This, in conjunction with the fact that water softeners alone do not remove most chemical or microbial contaminants, supports the participants' assertions that they used treatment devices solely to address the hardness and/or sulphur content of their well water, and not out of concerns for safety. Similarly, Levallois and colleagues found that only nine percent of 222 private water households in Quebec used treatment devices, with 11 percent of them doing so because of health concerns and the remainder for organoleptic reasons, like taste, odour and colour of their water [15]. The level and reasons for use of treatment devices among private water households in this study would appear to depend on the type of water system present, which could have implications for the best type of information to deliver to these different groups. Cistern owners for instance, might best benefit from information pertaining to the choice, use and maintenance of treatment devices, whereas well owners might benefit more from messages regarding the importance of water testing and what to do if a positive result is returned.

The current recommendation regarding routine testing of private water supplies in Ontario is to test water for indicator bacteria at least three times yearly, in the spring, summer and fall [20]. Testing of water for *E. coli *and total coliforms is free in the City of Hamilton, however, other test parameters are available at a cost to the owners of private water supplies. In this study, few participants regularly tested their well water, with most testing less frequently than provincial recommendations. This is comparable with results from other Canadian studies, which report that rural residents test their private water supplies intermittently, if at all [1,3]. It is important, however, to understand why residents choose not to test their water. Such data could be used to improve current public health education programs, encourage water testing and potentially help reduce the risk of disease from private water supplies. For instance, many participants in this study did not regularly test their water because their results had always been negative in the past. This may indicate the need to disseminate information regarding intermittent contamination and the changing quality of private water supplies. Many participants also said that they did not test their water because it looked and tasted normal; hence, they may benefit from being informed that many contaminants are odourless, colourless and tasteless [21]. Others said they were not aware of the need to test their water, which suggests the need for increased awareness of private water testing in general. One of the factors most contributing to the participants' decision not to test their water was the inconvenience of the testing process. If this was representative of the overall population, it would indicate the need to emphasize the importance of testing, and/or increase the convenience of the process, perhaps via a water sample pick-up service, or increased and more convenient sample-bottle pick-up and drop-off locations.

Inquiry as to residents' thoughts on how to encourage testing is also of great utility. By directly asking what would work to encourage private water owners to test their water, we are better equipped to design effective public health outreach activities. In this study, participants valued the creation of an educational program that would make water testing habitual among private water system owners. Given current Canadian recommendations regarding test frequency, a program that included public reminders with the changing of the seasons may be appropriate. Based on our results, a reminder campaign, released through a variety of public media outlets, designed to associate water testing with well-recognized dates may be appropriate. In Canada, such dates could be the first days of spring, summer and fall, or holidays like Easter (March or April), Father's Day (June) and Thanksgiving Day (October). Increasing the convenience of the process also appears to be warranted. Ideally, suitable funding would be made available to the appropriate health departments to implement such changes. Perhaps community groups or high-school community involvement curricula might organize water sample pick-up and drop-off programs. Similarly, the local health department could increase the number of service locations, particularly in the more rural areas of the City.

Another idea endorsed by participants was the creation of a results-sharing program that could be used by private water supply owners to learn the test results of private water supplies within their locality. While the need for confidentiality would obviously have to be addressed, participants felt that such a system would work in promoting water testing. This is also supported by virtue of the fact that learning of local water problems was the sole motivating factor behind some participants testing their water in the past. Perhaps creation of a system that provided basic test results of very general areas (for instance, "West Hamilton") would be useful. Further investigation of the testing behaviours and opinions of the general population, as well as the logistics of such a system, is required however, before such recommendations can be made.

This study highlighted areas of drinking water information that require increased dissemination in this population. Participants wanted more information on water testing, and participants in the 20–35 year-old age group said they were broadly lacking in knowledge and wanted more information regarding private water supplies in general. This suggests that the targeting of the younger generations may be warranted. Some participants also suggested a need to target residents new to rural areas, who may not otherwise be informed with respect to private water supplies; a point supported by other researchers [19,21]. In addition to their role in ensuring water quality as part of current regulations regarding home buying/selling, perhaps real estate agencies might collaborate with public health departments. For instance, they could disseminate informative flyers/other media regarding the importance of regular, on-going water testing to those purchasing homes with private water systems. Similarly, this information could be provided when property ownership changes and the deed to the home is transferred.

Some of the information that participants wanted is contained within a water well Best Management Practices Guide [20], which is made available by the health department in multiple community centers within the City of Hamilton. Unfortunately, participants in this study were unaware of its existence. Further, while informative, well-organized and illustrated, the guide is approximately 90-pages long and may therefore be overwhelming for some residents. Participants in this study said they wanted information to be clear, to the point, and distributed using other forms of media, like newspapers, radio and flyers distributed to their homes. If the results of this study are representative of the larger population, it would indicate the need for changes to water safety communication within the City of Hamilton. For instance, an effective program might include flyers or short radio announcements with general information that also directs residents to other sources of information, like a webpage or the Best Management Practices guide. This would also be a more effective way of targeting residents who are not actively seeking such information.

These focus groups allowed us to explore, in-depth, participants' perceptions of water from their private water supplies, including their self-identified need and desire for more information. We were able to gain this understanding with little interviewer influence, and were therefore privy to participant issues that we had not previously considered. While labour- and cost-intensive, the focus groups provided a detailed level of understanding of perceptions that we could not have been gained through quantitative survey alone. The ultimate goal of focus group research however, is to gain understanding, not to generalize. Thus, recommendations cannot be made solely on the results of this study, as the sample likely is not representative of the general population. This study has however, provided insight into the residents' perceptions of water from private supplies, including the vocabulary used by participants' to discuss the issues that were most important to them. Additionally, this information will be used to develop a survey instrument to investigate this population's perceptions on a larger scale. Our survey questions and categories are therefore more likely to reflect accurately the residents' empirical world, which will contribute to the validity of our survey instrument.

Despite their benefits, the focus groups are not without their limitations. Ideally, additional focus groups would have been conducted in order to ensure saturation of the data. Unfortunately, this was not possible due to budgetary limitations. Further, we had difficulty meeting target recruitment numbers, particularly in the younger age group, which may have contributed to selection bias. There are likely two main reasons for this. First, residents on private water systems are a minority in the population of the City of Hamilton. Second, our mapping system to identify residences with private water supplies had a predictive value of just 61 percent (Jones et al., unpublished), and was more likely to misclassify private system residences as having municipal water, particularly when they were near municipal water system boundaries, than vice versa. Hence, we may have inadvertently narrowed our selection pool, and increased the relative proportion of "very rural" residences. Given that only three focus groups could be conducted, selection criteria were limited and did not include ethnicity. The fact that only Caucasians attended the groups, as well as the requirement for participants to be English-speaking may have narrowed the transferability of the results. According to Statistics Canada 2001 census data of the City of Hamilton, however, approximately 97% of the population is able to converse in English; hence selection bias in this latter regard is likely to have been minimal. Future investigations would be strengthened via the inclusion of participants from a diversity of ethnic backgrounds. It is also possible that some participants responded to questions in a socially desirable manner, modifying their true responses as a result of other people in the room. However, we made every effort to prevent this type of bias by clarifying before the groups started that there were no "right or wrong" answers, and participants were encouraged to agree or disagree with one another as appropriate. As group knowledge of socio-economic status is also known to affect group interactions, we were careful to avoid any discussion of this prior to and during, the focus groups, as recommended [11,12]. Overall, the focus groups were non-confrontational and friendly in nature suggesting that participant influence in this manner was minimal.

## Conclusion

We performed an in-depth investigation of participants' perceptions of drinking water from private water supplies in the City of Hamilton, Ontario (Canada). Most participants, particularly those over the age of 35, were confident in the safety and quality of their water. There is some question however, as to whether these opinions are well informed. There was a high degree of uncertainty in discussing water testing, and most participants did not test their private water supplies with the frequency currently recommended. Further, where testing was done, it was for *E. coli *and total coliforms only, primarily via the free testing service. This may pose a public health risk, as contamination of private water supplies in various parts of Canada is well documented [1,3,4,15,16]. The results of this study show that participants want and need more information pertaining to private drinking water supplies, particularly with regard to water testing. They also suggest the need for changes to current information dissemination efforts in the population. Further, observed differences in the perceptions and needs of people on different private water systems and between different age groups may suggest the need for targeted public health strategies. If the results of this study are applicable to the general population, action on the part of public health officials, and potentially various levels of government, is necessary. A valid understanding of residents' perceptions, needs and concerns with respect to private drinking water supplies is integral to the development of effective public health strategic planning, public education programs and drinking water policy. These focus groups provided significant insight in this regard, but it is important that larger scale investigations of the perceptions of drinking water from private water supplies be performed.

## Competing interests

The author(s) declare that they have no competing interests.

## Authors' contributions

SJH conceived the project; AQJ designed the study, administered/moderated the focus groups, collected and analyzed the data, and drafted the manuscript; CED, KD, SEM, SAMc, DWT, SJH and EM provided critical feedback on the study design, the analyses and interpretation of results, as well as editorial comments on the manuscript. SEM also assisted with administration/moderation of the focus groups.

## Pre-publication history

The pre-publication history for this paper can be accessed here:



## References

[B1] Corkal D, Schutzman WC, Hilliard C (2004). Rural water safety from the source to the on-farm tap. J Toxicol Environ Health A.

[B2] Levallois P, Guevin N, Gingras S, Levesque B, Weber JP, Letarte R (1998). New patterns of drinking water consumption: results of a pilot study. Sci Total Environ.

[B3] Thompson TS (2003). General chemical water quality of private groundwater supplies in Saskatchewan, Canada. Bull Environ Contam Toxicol.

[B4] Strauss B, King W, Ley A, Hoey JR (2001). A prospective study of rural drinking water quality and acute gastrointestinal illness. BMC Public Health.

[B5] Raina PS, Pollari FL, Teare GF, Goss MJ, Barry DA, Wilson JB (1999). The relationship between E. coli indicator bacteria in well-water and gastrointestinal illnesses in rural families. Can J Public Health.

[B6] Auslander BA, Langlois PH (1993). Toronto tap water: perception of its quality and use of alternatives. Can J Public Health.

[B7] Lee S, Levy D, Hightower A, Imhoff B, EIP FoodNet Working Group (2002). Drinking water exposures and perceptions among 1998–1999 FoodNet survey respondents.

[B8] Levallois P, Grondin J, Gingras S (1999). Evaluation of consumer attitudes on taste and tap water alternatives in Quebec. Water Sci Tech.

[B9] AWWARF (American Water Works Association Research Foundation) (1993). Consumer Attitude Survey on Water Quality Issues.

[B10] Jones A, Dewey C, Dore K, Majowicz S, McEwen S, Waltner-Toews D Drinking water consumption patterns in a Canadian community. J Water Health.

[B11] Krueger RA, Casey MA (2000). Focus Groups: a practical guide for applied research.

[B12] Morgan DL, Krueger RA (1997). The Focus Group Kit.

[B13] Taylor-Powell E, Renner M (2003). Analyzing Qualitative Data.

[B14] Bruce-Grey-Owen Sound Health Unit (2000). The Investigative Report of the Walkerton Outbreak of Waterborne Gastroenteritis.

[B15] Levallois P, Theriault M, Rouffignat J, Tessier S, Landry R, Ayotte P, Girard M, Gingras S, Gauvin D, Chiasson C (1998). Groundwater contamination by nitrates associated with intensive potato culture in Quebec. Sci Total Environ.

[B16] Frank R, Chapman N, Johnson R (1991). Survey of farm wells for nutrients and minerals, Ontario, Canada, 1986 and 1987. Bull Environ Contam Toxicol.

[B17] Rutter M, Nichols GL, Swan A, De Louvois J (2000). A survey of the microbiological quality of private water supplies in England. Epidemiol Infect.

[B18] Borchardt MA, Bertz PD, Spencer SK, Battigelli DA (2003). Incidence of enteric viruses in groundwater from household wells in Wisconsin. Appl Environ Microbiol.

[B19] Said B, Wright F, Nichols GL, Reacher M, Rutter M (2003). Outbreaks of infectious disease associated with private drinking water supplies in England and Wales 1970–2000. Epidemiol Infect.

[B20] Agriculture and Agri-Food Canada (2003). Best Management Practices: Water Wells.

[B21] Simpson H (2004). Promoting the management and protection of private water wells. J Toxicol Environ Health A.

